# The correlation analysis between preoperative cerebral small vessel disease and carotid plaque echo characteristics

**DOI:** 10.3389/fneur.2025.1625480

**Published:** 2025-09-26

**Authors:** Yanqing Zhou, Pianpian Yan, Shengwen Guo, Yiting Huang, Lulu Jiang

**Affiliations:** Department of Anesthesiology, Xiamen Cardiovascular Hospital of Xiamen University, School of Medicine, Fujian Branch of National Clinical Research Center for Cardiovascular Diseases, Xiamen, China

**Keywords:** carotid plaque, cerebral small vessel disease, ultrasound, stroke, ultrasound imaging

## Abstract

**Purpose:**

This study explores the correlation between carotid plaques and cerebral small vessel disease (CSVD).

**Method:**

A total of 167 patients were divided into CSVD (*n* = 119) and Non-CSVD (*n* = 48) groups. Various clinical variables, including body mass index (BMI), coronary artery disease, hypertension, and carotid plaque echo characteristics, were evaluated using multivariate logistic regression analysis.

**Results:**

The analysis revealed that carotid isoechoic plaques (OR = 2.139, 95% CI 1.421–3.219, *P* < 0.001) and hypoechoic plaques (OR = 1.687, 95% CI 1.206–2.359, *P* = 0.002) are independent risk factors for CSVD. Other variables such as BMI, coronary artery disease, hypertension, and hyperlipidemia did not reach statistical significance.

**Conclusion:**

The presence of carotid isoechoic and hypoechoic plaques significantly increases the risk of CSVD, highlighting the importance of carotid imaging in assessing stroke risk and guiding clinical management.

## Introduction

Perioperative stroke is a significant and devastating complication, particularly prevalent in patients undergoing cardiac and major vascular surgeries, with reported incidences ranging from 2% to 10% (1). This condition poses a major challenge to surgical outcomes, leading to increased morbidity and mortality. Among the elderly population, cerebral small vessel disease (CSVD) is a highly common neurological disorder and a leading cause of stroke, contributing to an estimated 20% to 30% of all strokes in this demographic (2–4). The pathophysiology of CSVD is often linked to various vascular risk factors, including hypertension, diabetes, and dyslipidemia, which exacerbate small vessel damage and promote ischemic events (5, 6). Recent studies have identified carotid plaques as significant markers of atherosclerosis, with independent associations with both stroke and cardiovascular incidents (7). It has suggested that the presence of carotid plaques can increase stroke risk by up to 50%, emphasizing their role as critical indicators in cerebrovascular assessment (8). Furthermore, the characteristics of these plaques, particularly their echogenicity, can provide valuable prognostic information regarding vascular health (9).

Ultrasound imaging serves as an effective, non-invasive tool for evaluating carotid arteries, offering advantages such as the absence of ionizing radiation and the ability to assess plaque morphology directly (10, 11). Given that carotid arteries are located superficially, ultrasound can yield detailed insights into plaque characteristics, which may correlate with the presence and severity of CSVD (12, 13).

This study aims to investigate the echo characteristics of carotid plaques through ultrasound to determine their correlation with CSVD and assess associated independent risk factors. By elucidating these relationships, we seek to contribute to a more nuanced understanding of CSVD, ultimately enhancing risk stratification and informing clinical management strategies in at-risk populations.

## Method and material

### Study sample

**Figure 1:**
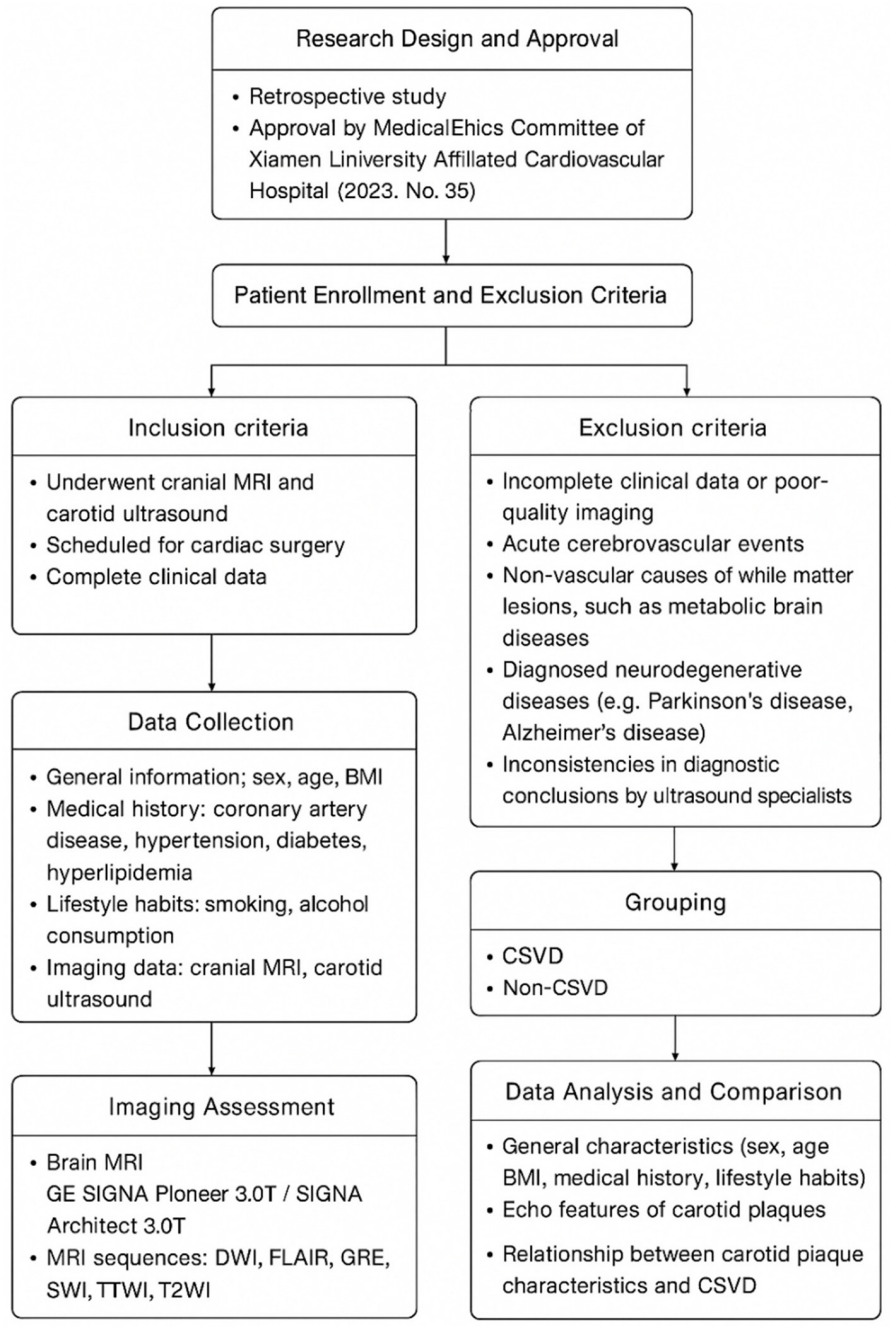
Flowchart of study design, patient selection, imaging assessment, grouping, and data analysis in relation to carotid plaque characteristics and CSVD.

This retrospective study involved the continuous collection of data from inpatients scheduled for cardiac surgery at Xiamen University Affiliated Cardiovascular Hospital between May 2019 and September 2023. A total of 167 patients were enrolled, including 93 males (55.7%) and 74 females (44.3%), with an age range of 40 to 85 years (mean age: 61.3 ± 8.6 years). Inclusion criteria required patients to have undergone both cranial MRI and carotid ultrasound at our institution. All patients were scheduled for cardiac surgery, and carotid ultrasound was performed as part of routine preoperative screening to assess cerebrovascular risk. Preoperative screening considered patients' cardiovascular risk factors, including hypertension, diabetes, and hyperlipidemia. Exclusion criteria included: (1) incomplete clinical data or poor-quality imaging; (2) acute cerebrovascular events; (3) non-vascular causes of white matter lesions, such as metabolic brain diseases; (4) diagnosed neurodegenerative diseases (e.g., Parkinson's disease, Alzheimer's disease); and (5) inconsistencies in diagnostic conclusions by ultrasound specialists (Figure 1). Although routine head/neck CTA (CT angiography) was not performed, patients with ≥50% carotid stenosis or abnormal ultrasound results were further evaluated with imaging studies (e.g., CTA or MRI angiography) as clinically indicated. Given the high prevalence of intracranial stenosis in the Asian population, both clinical background and carotid plaque characteristics were considered to comprehensively assess cerebrovascular health and small vessel disease burden. The study was approved by the Medical Ethics Committee of Xiamen University Affiliated Cardiovascular Hospital (2023, ethical approval number 35).

### Data collection and grouping

Clinical data were collected from patients, including general information such as sex, age, body mass index (BMI), history of coronary artery disease, history of hypertension, history of diabetes, history of hyperlipidemia, as well as smoking and alcohol consumption histories. Additionally, cranial MRI and carotid ultrasound images were obtained. Based on the cranial MRI results, the study participants were classified into two groups: those with cerebral small vessel disease (CSVD) and those without cerebral small vessel disease (Non-CSVD). The differences in general characteristics and the echo features of carotid plaques between the two groups were then compared to elucidate the relationship between carotid plaque characteristics and CSVD.

### Diagnostic criteria

Cranial MRI was conducted using the GE Healthcare SIGNA Pioneer 3.0T and SIGNA Architect 3.0T superconducting magnetic resonance imaging systems. The imaging sequences included axial diffusion-weighted imaging (DWI), T2 fluid-attenuated inversion recovery (FLAIR), T2^*^-weighted gradient-recalled echo (GRE), susceptibility-weighted imaging (SWI), as well as T1-weighted imaging (T1WI) and T2-weighted imaging (T2WI) (14). The established radiological markers for the diagnosis of cerebral small vessel disease included recent small subcortical infarcts (RSSI), vascular lacunes, vascular-related white matter hyperintensities (WMHs), enlarged perivascular spaces (EPVS), cerebral microbleeds (CMBs), and brain atrophy (15). These criteria facilitate a comprehensive assessment of the structural changes associated with CSVD, thereby enhancing diagnostic accuracy.

### Ultrasound examination

Ultrasound examinations were performed using the Philips EPIQ 7 diagnostic ultrasound system, equipped with linear array transducers operating at frequencies ranging from 5.0 to 12.0 MHz. Two-dimensional ultrasound combined with color Doppler ultrasound was utilized to conduct both longitudinal and transverse scans of the carotid arteries, subclavian arteries, and vertebral arteries. The degree of carotid stenosis and plaque characteristics were evaluated following the Mannheim Carotid Intima-Media Thickness and Plaque Consensus (16). Based on the echo characteristics of the vascular wall, carotid plaques were categorized into three types according to the echogenicity of the plaque contents: (1) hypoechoic plaques: those with echogenicity lower than that of the intima-media layer of the vascular wall; (2) isoechoic plaques: those with echogenicity consistent with that of the intima-media layer; and (3) hyperechoic plaques: those with echogenicity equal to or slightly higher than that of the adventitial layer of the vascular wall (17). All examinations were conducted by operators of the same professional qualification level to ensure consistency and reliability in the assessment.

### Statistical analysis

Data was conducted by SPSS 29.0. Initially, univariate analyses were performed to compare the general characteristics and carotid echo features between the two patient groups. Continuous data conforming to a normal distribution were expressed as mean ± standard deviation (x ± s) and analyzed using independent samples *t*-tests. Categorical data were presented as counts (percentage) and analyzed using the χ^2^ test or Fisher's exact test as appropriate. All tests were two-tailed, with a significance level set at α = 0.05. Variables demonstrating statistically significant differences (*P* < 0.05) in clinical characteristics between the two groups were subsequently subjected to multivariate logistic regression analysis to identify potential risk factors for cerebral small vessel disease. *P* < 0.05 was considered statistically significant.

## Results

### Comparison of general characteristics between the two groups

Initially, we analyzed the differences in general characteristics between the two patient groups. The results indicated no statistically significant differences in sex, age, diabetes, hyperlipidemia, smoking, or alcohol consumption (*P* > 0.05). However, the BMI of the CSVD group was higher than that of the Non-CSVD group [(24.1 ± 3.4) kg/m^2^ vs. (22.8 ± 3.2) kg/m^2^; t = 2.237, *P* = 0.027]. Additionally, the prevalence of coronary artery disease and hypertension was significantly higher in the CSVD group compared to the Non-CSVD group (both *P* < 0.05; Table 1).

**Table 1:** The general characteristics between the two groups.

**Characteristic**		**Non-CSVD (*n* = 48)**	**CSVD (*n* = 119)**	***P***
Sex	Male	22	57	0.512
	Female	26	62	
Age		62.0 ± 8.5	61.0 ± 8.7	0.350
Diabetes	No	38	84	0.150
	Yes	10	35	
Hyperlipidemia	No	33	64	0.250
	Yes	15	55	
Smoking history	No	28	54	0.180
	Yes	20	65	
Drinking history	No	30	47	0.065
	Yes	18	72	
BMI		22.8 ± 3.2	24.1 ± 3.4	0.027
Coronary artery disease	No	30	51	0.026
	Yes	18	68	
Hypertension	No	36	65	0.015
	Yes	12	54	

### Comparison of carotid plaque echo characteristics between the two groups

Understanding the echo characteristics of carotid plaques in different patient populations is crucial for assessing stroke risk and guiding treatment strategies. In this study, we found that the proportion of patients in the CSVD group without carotid plaques was significantly lower than that in the Non-CSVD group (*P* < 0.001). Additionally, the prevalence of hypoechoic and isoechoic plaques was significantly higher in the CSVD group compared to the Non-CSVD group (both *P* < 0.05; Table 2).

**Table 2:** Comparison of carotid plaque echo characteristics between the two groups.

**Classification**	**CSVD (*n* = 119)**	**Non-CSVD (*n* = 48)**	***P***
Type I	45 (26.95%)	21 (12.57%)	0.295
Type II	98 (58.68%)	47 (28.14%)	0.004
Type III	110 (65.87%)	48 (28.74%)	0.043

Type I: hyperechoic plaques; Type II: isoechoic plaques; Type III: hypoechoic plaques.

### Analysis of risk factors for CSVD

In the analysis of the aforementioned comparisons, variables with *P* < 0.05, including BMI, coronary artery disease, hypertension, hypoechoic plaques, and isoechoic plaques, along with other clinically significant variables identified by the researchers such as hyperlipidemia, were included in a multivariate logistic regression model. The results indicated that carotid isoechoic plaques (OR = 2.139, 95% CI 1.421–3.219, *P* < 0.001) and carotid hypoechoic plaques (OR = 1.687, 95% CI 1.206–2.359, *P* = 0.002) were independent risk factors for CSVD (Table 3).

**Table 3:** Analysis of risk factors for CSVD patients.

**Variable**	**Regression coefficient (β)**	**Standard error (SE)**	**Wald χ^2^**	***P***	**OR**	**95% CI**
Carotid isoechoic plaques	0.757	0.180	17.81	<0.001	2.139	1.421 – 3.219
Carotid hypoechoic plaques	0.525	0.183	8.79	0.002	1.687	1.206 – 2.359
BMI	0.200	0.150	1.54	0.215	1.221	0.900 – 1.651
Coronary artery disease	0.350	0.200	3.00	0.083	1.419	0.951 – 2.115
Hypertension	0.300	0.180	2.52	0.113	1.349	0.930 – 1.949
Hyperlipidemia	0.250	0.190	1.82	0.178	1.284	0.890 – 1.849

## Discussion

CSVD is a significant contributor to vascular cognitive impairment and stroke, particularly in the aging population. The association between CSVD and various cardiovascular risk factors has been well established in the literature (18). As the prevalence of CSVD increases, understanding its risk factors and underlying pathophysiology becomes essential for effective prevention and management strategies (19). This study investigated the relationship between carotid plaque characteristics and the occurrence of CSVD, contributing to the growing body of evidence regarding cerebrovascular health.

Herein, we found no statistically significant differences in demographic factors such as sex, age, or common comorbidities like diabetes and hyperlipidemia between the CSVD and Non-CSVD groups. However, the CSVD group exhibited a significantly higher body mass index (BMI) and greater prevalence of coronary artery disease and hypertension. This aligns with findings from previous studies indicating that obesity and hypertension are strong independent risk factors for CSVD and related cerebrovascular events (20–22). Notably, these findings reinforce the importance of monitoring and managing these risk factors in at-risk populations.

Furthermore, our analysis of carotid plaque echo characteristics revealed that the proportion of patients without carotid plaques was significantly lower in the CSVD group compared to the Non-CSVD group. Additionally, the prevalence of hypoechoic and isoechoic plaques was higher in the CSVD group. These findings support existing literature that associates plaque characteristics with cerebrovascular risk. For instance, hypoechoic plaques have been identified as more unstable and prone to rupture, leading to ischemic events (23–25). Furthermore, studies have shown that may indicate a higher degree of lipid content, correlating with an increased isoechoic plaques risk of stroke (26).

Multivariate logistic regression analysis in our study highlighted that both carotid isoechoic plaques and hypoechoic plaques were independent risk factors for CSVD, with odds ratios suggesting a significant association. This is consistent with previous research, which has identified similar echogenic characteristics as predictors of cerebrovascular diseases. For example, a study by Sztajzel et al. (27) demonstrated that plaque echogenicity is a crucial determinant of cerebrovascular risk, reinforcing the utility of ultrasound in assessing vascular health.

In conclusion, our findings underscore the critical role of carotid plaque characteristics in evaluating the risk of CSVD. The relationship between hypoechoic and isoechoic plaques and CSVD emphasizes the need for thorough vascular assessments in patients at risk. By integrating these evaluations into routine clinical practice, we can enhance early detection and management strategies, potentially mitigating the impact of CSVD on patient outcomes.

## Study limitation

This study has several limitations. It primarily focuses on patients undergoing cardiac surgery, which may limit the generalizability of the findings to broader populations, especially those without existing cardiovascular conditions. Additionally, while factors such as BMI, coronary artery disease, and hypertension were considered, the primary focus was on the relationship between plaque echogenicity and cerebral small vessel disease (CSVD). As a result, not all potential confounding variables were fully adjusted for. Future research should address a wider range of factors and explore more diverse populations to more accurately assess the independent impact of plaque echogenicity on CSVD and stroke risk.

## Data Availability

The raw data supporting the conclusions of this article will be made available by the authors, without undue reservation.
